# Dengue Virus Serotype 2 Cosmopolitan C Genotype Reemerges With a New Strain in Southwest Region of Bangladesh

**DOI:** 10.1155/tbed/8275099

**Published:** 2025-03-06

**Authors:** Md. Ali Ahasan Setu, Prosanto Kumar Das, Toukir Ahammed, Shuvo Saha, Adib Hasan, Shishir Kumar P. K., Samiran Das, Tanvir Ahamed, K. M. Amran Hossain, Hassan M. Al-Emran, M. Anwar Hossain, Iqbal Kabir Jahid

**Affiliations:** ^1^Department of Microbiology, Jashore University of Science and Technology, Jashore, Bangladesh; ^2^Genome Centre, Jashore University of Science and Technology, Jashore, Bangladesh; ^3^Department of Genetic Engineering and Biotechnology, Jashore University of Science and Technology, Jashore, Bangladesh; ^4^Department of Nutrition and Food Technology, Jashore University of Science and Technology, Jashore, Bangladesh; ^5^Department of Physiotherapy and Rehabilitation, Jashore University of Science and Technology, Jashore, Bangladesh; ^6^Department of Biomedical Engineering, Jashore University of Science and Technology, Jashore, Bangladesh

**Keywords:** Bangladesh, cMDS analysis, Cosmopolitan C, dengue serotype 2, genetic variation, phylogenetic tree

## Abstract

In 2023, the dengue virus (DENV) outbreak infected over 0.3 million cases and 1500 deaths in Bangladesh. Our study conducted serotyping and genomic surveillance in four districts of Southwest Bangladesh between September and October 2023. The surveillance data from 2019 to 2023 extracted from the Directorate General of Health Services in Bangladesh indicated a significant increase of Dengue infections in 2023, particularly during September–November. The two-layered hypothesis examination confirmed that, despite endemic months, 2023 dengue outbreak had a higher morbidity rate compared to previous years (2019–2022) in the southwest of Bangladesh. Serotyping using RT-PCR and E gene sequence analysis of 25 randomly selected positive samples reveals that DENV-2 was the dominant serotype circulating in this region during the study period. Genomic analysis (phylogenetic analysis and classical multidimensional scaling [cMDS]) exposed a new strain of DENV-2, classified under Cosmopolitan genotype within C clade, distinct from previous Bangladeshi strains until 2022. This strain, possibly migrating from India, might have emerged during the COVID-19 pandemic years and exhibited higher morbidity rates, thus challenging our existing mitigation strategies. This investigation provides valuable insights for public health interventions and underscores the importance of continuous genomic surveillance in managing dengue outbreaks.

## 1. Introduction

Antigenically distinct dengue virus serotypes 1 to 4 (DENV-1 to -4) are further subdivided into multiple genotypes based on the sequence variations in envelope (E) gene and named according to their geographic regions such as Asian I, Asian II, Asian/American, American, Cosmopolitan, and sylvatic. However, some genotypes have spread all over the world and substantially contributed to the global dengue burden and mortality. Cosmopolitan genotype of DENV-2 is the most widespread and genetically heterogeneous of all [1]. In Bangladesh, although sequence data are limited, DENV-2 was prevalent in 2017 and 2018, whereas DENV3 was prevalent in 2019 until August 2022 [2]. However, there was no report published on circulating DENV serotypes or genotypes data about ongoing 2023 outbreak in Bangladesh, where more than 1500 died during the outbreak [3]. Thus, monitoring of transmission dynamics faces a challenge of implementation of effective DENV mitigation strategies. This study endeavors to conduct genomic surveillance, providing valuable insights into the genotypes in the southwest region of Bangladesh.

## 2. Methods and Materials

### 2.1. Epidemiological Analysis of Morbidity Trends

As part of the study, DENV surveillance data spanning 30 months (2019–2023) were retrieved from the Directorate General of Health Services (DGHSs) to perform morbidity trend analysis (https://dashboard.dghs.gov.bd/pages/heoc_dengue_v1.php). The cases were analyzed through Statistical Package of Social Sciences (SPSSs) version 20. Normality test was performed by Kolmogorov–Smirnov test and Shapiro–Wilk test and found a non-normal distribution (*p*  < 0.0001). Descriptive analysis was presented by median and interquartile range (IQR). Our first hypothesis was the trend of seasonality of morbidity, we performed Kruskal–Wallis *H* test; the second hypothesis was increased morbidity in 2023 compared to 2019–2023, examined by Mann–Whitney *U* test [4]. The morbidity trend analysis was presented through a line graph and statistically tested by Mann–Kendall trend analysis [5]. The alpha value of the study was *p*  < 0.05.

Concurrently, a cross-sectional survey was undertaken to examine dengue-positive cases occurring between September and October 2023 in four districts of Southwest Bangladesh: Jashore, Magura, Narail, and Jhenaidah. Approval for the study was obtained from the Ethical Review Committee (ERC) of Jashore University of Science and Technology (JUST), Bangladesh (ERC no: ERC/FBST/JUST/2023-184). All procedures were conducted in accordance with applicable guidelines and regulations at the Genome Centre, JUST.

### 2.2. Sample Collection, Preliminary Screening, and Serotyping

During September–October 2023, 600 suspected dengue fever patients (both hospitalized and outdoors) were selected as the study subjects. The participants were informed about the study and interviewed through a questionnaire to obtain sociodemographic and health-related data. After obtaining informed written consent from the subjects, left-over blood samples were collected from the pathology labs of different government hospitals and private diagnostic centers. The blood samples were transported immediately to the Genome Centre under stringent cold-chain conditions, maintaining a temperature range of 2°C–8°C using insulated containers with ice packs to ensure sample integrity. Preliminary screening was conducted using the Dengue NS1 Rapid Test Kit (Hangzhou Frenovo Biotech Co., Ltd., China). Viral RNA of NS1-positive samples was then extracted using the QIAamp Viral RNA Mini Kit (Cat. No. 52906, Qiagen, Hilden, Germany). After extraction, the DENV RNA was serotyped using Molaccu DENV qPCR Detection Kit (Cat. No. 01.09.21.16.32.02, Zybio Inc., China) in qTOWER^3^G RT-PCR instrument (Analytic Jena, Germany) following manufacturer instructions.

### 2.3. Partial Genome Sequencing and Data Analysis

Based on a cycle threshold (Ct) value of ≤26, representative samples from each week of the study period were selected for sequencing. cDNAs were prepared using SuperScript III First-Strand Synthesis kit (InvitrogenTM, Thermo Fisher Scientific, USA). Two overlapping fragments of the DENV-2 E gene were amplified by using d2s1C-CC (AGTTGTTAGTCTACGTGGACCGACA), DEN2 CC- 1R (AGCACCATCTCATTGAAGTCGAGG), DEN2 CC 1F (CAAACACTCCATGG TAGACAGAGG), and d2a18-CC (TCCCRCTGCCACATTTCARTTCTTT) primers [6] and verified through Agarose gel electrophoresis. Sanger sequencing was performed on randomly selected positive samples of each week throughout the study period in Applied Biosystems SeqStudio genetic analyzer using BigDye Terminator v3.1 as per the optimized protocol [7]. All forward and reverse ab1 files from the sequencing run were simultaneously quality trimmed in Chromas and assembled in Unipro UGENE followed by genotyping in GISAID Dengue Server (https://www.epicov.org/epi3/frontend#1754f5). Additional nonsylvatic DENV 2 genome sequences were retrieved from the publicly available database (NCBI Virus) and aligned with the sequenced data of this study using MAFFT.

A dataset of 594 sequences of DENV-2 E gene (1485bp in length) comprising 25 samples from the clinical study (2023) and 569 samples from NCBI virus database (2000–2023) was used for phylogenetic analysis. We took all five genotypes (Asian I; *n* = 4, Asian II; *n* = 2, Asian-American; *n* = 3, American; *n* = 1, Cosmopolitan; *n* = 584) for this analysis. Maximum likelihood method was employed to build phylogenetic tree using IQ-TREE2. The best-fit model TIN2+F+I+G4 was determined through ModelFinder. Subsequently, an ultrafast bootstrap with 100,000 replicates was calculated [8, 9]. The phylogenetic tree was visualized using i-Tol [10].

Multidimensional scaling (MDS) was employed to visualize the relationship among different sets of sequences (variants and clades) depending on the distance matrix [11]. In this study, distance matrix of DENV-2 Cosmopolitan C sequences was created using custom R script (library used: stats, ape, and ade4) by calculating pairwise genetic distances (TN93 model). Then, we performed hierarchical clustering to build a dendrogram and cut the dendrogram to define clusters (height parameter, *h* = 0.025; global, *h* = 0.015; BD). Following this step, the pairwise genetic distances were converted into Euclidean distances using the classical multidimensional scaling (cMDS) method (cmdscale function in R version 4.3.0).

## 3. Results

**Figure 1:**
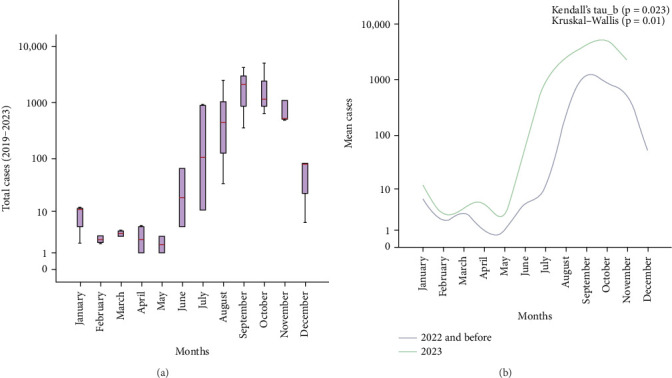
(A) Seasonal variation of dengue cases of southern part of Bangladesh (2019–2023). Colored box indicates Q2–Q3, red line indicates median (*p*  < 0.05). (B) Month-wise Mann–Kendall trend analysis (2019–2022 versus 2023) (data source: https://dashboard.dghs.gov.bd/pages/heoc_dengue_v1.php).

The median morbidity cases of dengue between 2019 and 2022 were 34 (IQR 487) and in 2023 were 64 (IQR 2478). The highest median cases were observed in September (2129), October (1134), and November (512).  Figure 1A illustrates the overall cases between 2019 and 2023 according to the distribution of months. The seasonal increase in cases (September–November) was found significant (*p*  < 0.05), suggesting its predictive potential as a contributing factor. Further analysis found a significant (*p*  < 0.05) mean difference in yearly cases in 2023 compared to 2019–2022. Finally, a Mann–Kendall trend analysis comparing the monthly cases from 2019 to 2022 to those of 2023 revealed a statistically significant (*p*  < 0.05) increasing trend, as shown in Figure 1B.

Out of the 600 suspected dengue cases, 145 were confirmed positive by the NS1 antigen test. Among these, 143 cases were identified as DENV serotype 2 through RT-PCR analysis. Based on a Ct value of ≤26, 25 samples were selected for sequencing.

**Figure 2:**
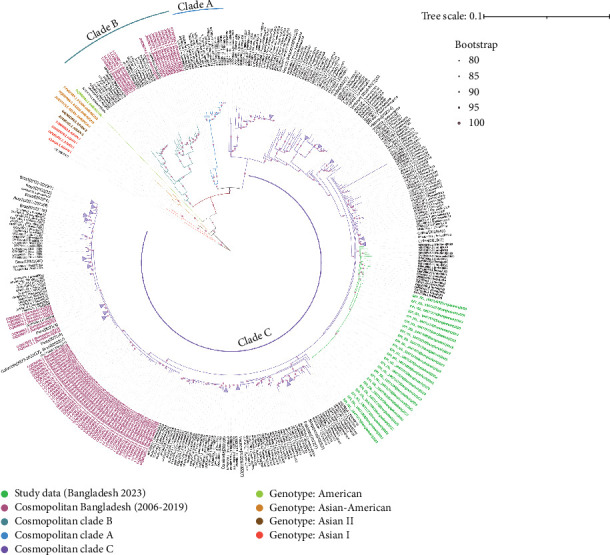
A maximum likelihood phylogenetic tree was built using the W-IQ-TREE software. ModelFinder was employed to select the optimal model, and ultrafast bootstrapping (UFBoot) with 100,000 replicates was utilized. The TIN2+F+I+G4 model was determined to be the most suitable fit for the analysis. The dataset includes DENV-2 E-gene sequences obtained in the current study, highlighted in green for the year 2023. Additionally, DENV-2 sequences from Bangladesh spanning the years 2000–2022 are represented in pink. The Cosmopolitan clade (A, B, and C) is demarcated by arc and corresponding colors. Each sample name comprises the accession number, country, and reported year of the sequence. Branches on the phylogenetic tree are annotated with UFBoot support values, with values exceeding 90% being highlighted.

**Figure 3:**
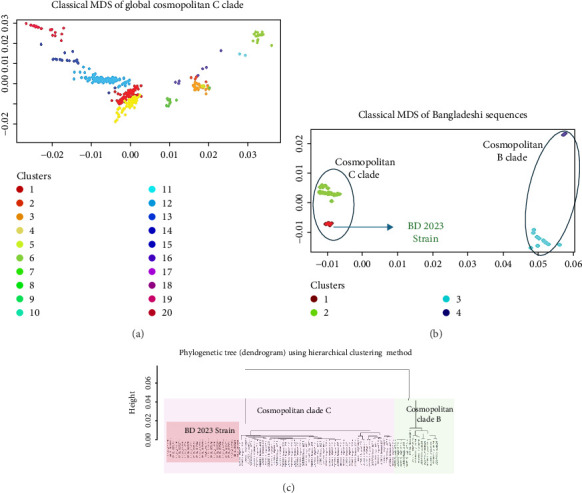
Multidimensional scaling (MDS) representation of DENV-2 Cosmopolitan sequences. Panel (A) illustrates the cMDS of global DENV-2 Cosmopolitan C sequences, revealing 20 distinct clusters. Here BD 2023 strain has clustered with neighboring countries sequences (red clusters). Panel (B) illustrates the cMDS of Bangladeshi Cosmopolitan clades revealed four clusters, two of each for Cosmopolitan B and C clades (within circle). Red cluster indicates the emergence of BD 2023 strain. Panel (C) represents the hierarchical clustering of Bangladesh clades.

All 594 sequences used in this study were genotyped using GISAID Dengue server. All 25 sequences of this study clustered together in clade C of Cosmopolitan genotype, as shown in Figure 2. DENV-2 Bangladeshi strains from 2006 to 2017 (*n* = 16) were Cosmopolitan within B clade, from 2017 to 2018 (*n* = 55) were Cosmopolitan C clade, whereas in 2017, the strains were in both Clades B and C (Figure 2). Previous study showed that Bangladesh had no Cosmopolitan sequences from 2020 to 2022 [12]. The Bangladeshi 2023 strain we found in this study belongs to Cosmopolitan C clade. This Bangladeshi 2023 strain of Cosmopolitan C is genetically distant from previous Bangladeshi strains. MDS was conducted on two distinct datasets: global sequences representing the Cosmopolitan C clade and local sequences from Bangladesh encompassing all Bangladeshi clades. This analysis revealed the formation of four distinct clusters, as shown in Figure 3C. Notably, two clusters were associated with the Cosmopolitan B clade, while the remaining two clusters were attributed to the Cosmopolitan C clade. Our BD 2023 sequences were identified to belong to the Cosmopolitan C clade, as indicated by the red circle in Figure 3B.

Subsequently, MDS analysis of pairwise genetic distances among global sequences of the Cosmopolitan C clade uncovered 20 clusters in two-dimensional space. Notably, within the cluster delineated by the red circle, the emergence of a new strain in Bangladesh was confirmed, as depicted in Figure 3A. Hierarchical clustering tree of global sequences can be found in the Supporting Information section. These findings underscore the local genetic dynamics shaping the evolution of strains within the Cosmopolitan C clade. This BD 2023 strain clustered with the neighboring country (India, China, Thailand, Sri Lanka, Vietnam, and Cambodia) sequences.

## 4. Discussion

Dengue poses a significant public health challenge, particularly in urban and semiurban regions, where the majority of outbreaks have been documented [13]. The global incidence of dengue has increased 30-fold over the past five decades and Bangladesh has experienced annual dengue outbreaks since 2000, with a concerning trend of increasing severity in recent years [14, 15].

Our investigation of the 2023 dengue outbreak in the southwest region of Bangladesh revealed a higher morbidity rate compared to previous years (2019–2022), despite ongoing endemicity. This outbreak was characterized by an earlier seasonal rise in cases, rapid geographical spread, and a particularly high fatality rate [16]. Our study identified a DENV-2 Cosmopolitan strain within the C clade as the dominant strain responsible for the 2023 outbreak. Other studies have also reported that, in 2023, Dengue serotype 2 replaced the previously predominant Dengue serotype 3 [17, 18]. This strain displayed statistically significant trends of increased cases between September and November, coinciding with the postmonsoon season.

Previous outbreaks in Bangladesh (2017 and 2018) were also caused by the DENV-2 Cosmopolitan genotype within the B and C clades [12]. However, the 2023 strain showed no significant similarity to previously documented Bangladeshi or global C clade strains (2017, 2018, and 2019), suggesting the emergence of a new strain. This new strain exhibited greater genetic similarity to isolates from India (*n* = 12, 2018–2021), China (*n* = 1, 2022), Indonesia (*n* = 1, 2020), Thailand (*n* = 2, 2020), and the USA (*n* = 3, 2023) (Figure 2).

In our preliminary analysis, we initially posited that the 2023 Bangladeshi sequences belonged to a new subclade of Cosmopolitan C, based on phylogenetic tree topology [19]. However, it became apparent that they were not well-suited for subspecies classification. Subsequent analysis using MDS revealed four clusters (with a genetic distance threshold of *h* = 0.015; representing 1.5% pairwise genetic distance used to partition the hierarchical dendrogram) within local sequences (two of each for Cosmopolitan B and C clades) and 20 clusters encompassing global sequences. Notably, the 2023 Bangladeshi sequences (BD 2023) clustered within the Cosmopolitan C clade. This finding suggests the emergence of a new strain within Bangladesh can be driven by local genetic dynamics such as the prevalence of certain *Aedes* mosquito species, regional variations in viral evolution, and the impact of previous outbreaks. Studies have shown that local transmission dynamics, including vector population density and environmental factors, can significantly influence the genetic evolution of DENVs [20, 21]. The clustering of this strain with isolates from neighboring countries highlights Bangladesh's potential role as a hotspot for DENV strain emergence. The study area's proximity to major Indian land ports (Petropole and Benapole) raises the possibility of cross-border transmission, supported by the high degree of genetic similarity (99% bootstrap support) observed between Bangladeshi isolates from our study and those identified in India during 2022.

*Aedes aegypti* mosquitoes and, to a lesser extent, *Aedes albopictus* mosquito are the primary vectors for DENV transmission and thrive in Bangladesh's climate, which is becoming increasingly favorable for vector-borne diseases [22]. Several factors likely contributed to the elevated case numbers in the 2023 outbreak. These include neighboring country's outbreaks timing and patterns of holiday travel [23] and climate variability [24]. Additionally, antibody-dependent enhancement (ADE) may have played a role in altering transmission dynamics [25]. ADE occurs when preexisting subneutralizing antibodies from a previous dengue infection facilitate viral entry into host cells, leading to enhanced viral replication and severe disease outcomes [26]. Between 2019 and August 2022, DENV-3 was the predominant circulating serotype; however, DENV-2 emerged as the dominant strain during the 2023 epidemic. Notably, 17% of patients developed severe dengue, while 89% of those with nonsevere cases presented at least one warning sign, highlighting the heightened clinical severity associated with the outbreak [27].

Bangladesh's healthcare system was already burdened by the severe third wave of COVID-19. The surge in dengue cases during this period created a “double burden” on the healthcare infrastructure [28]. Under such circumstances, genomic surveillance becomes pivotal, offering valuable insights into genetic variations and evolutionary trends. This information is vital for guiding public health interventions and vaccine development.

The emergence of a new strain and its potential impact on the ongoing outbreak further highlights the need for research on coinfection and their potential effects on viral evolution and pathogenicity, and the dynamics of the cross-border spread.

## Data Availability

The datasets and R scripts mentioned in this study are available on GitHub. The reader(s) can access them via the following link: https://github.com/aasetu/Dengue_project.

## Supporting Information

Supporting Information Supporting Information S1 includes the dendrogram of global Cosmopolitan C sequences, while S2 and S3 contain tables presenting the global and local cMDS clusters, respectively.
