# Supplementary material for: Dengue Virus Serotype 2 Cosmopolitan C Genotype Reemerges With a New Strain in Southwest Region of Bangladesh
Source: Transbound Emerg Dis. 2025 Mar 6;2025:8275099. doi: 10.1155/tbed/8275099 (PMC12016812; doi:10.1155/tbed/8275099)
Supplement: Supporting Information — S1 includes the dendrogram of global Cosmopolitan C sequences, while S2 and S3 contain tables presenting the global and local cMDS clusters, respectively. [file 8275099.f1.zip › S2_cluster_global_cosmopolitan_c.pdf]

Accession	Country	Year	Cluster
OR771143.1	USA	2023	1
OR771194.1	USA	2023	1
OR771196.1	USA	2023	1
MN444617.1	Laos	2019	1
KT232052.1	Nepal	2013	1
KY495802.1	India	2016	1
MN913511.1	China	2019	1
MT705576.1	China	2019	1
MT705577.1	China	2019	1
MT705578.1	China	2019	1
MT705579.1	China	2019	1
MT705580.1	China	2019	1
MT705581.1	China	2019	1
MT705585.1	China	2019	1
MT705586.1	China	2019	1
MT705587.1	China	2019	1
MT705588.1	China	2019	1
MT705592.1	China	2019	1
MT705596.1	China	2019	1
MT705598.1	China	2019	1
MT705600.1	China	2019	1
MT705601.1	China	2019	1
MT705602.1	China	2019	1
MT705607.1	China	2019	1
MT705611.1	China	2019	1
MT705612.1	China	2019	1
MT705613.1	China	2019	1
MT705614.1	China	2019	1
MT705615.1	China	2019	1
MT754367.1	China	2019	1
MT754368.1	China	2019	1
MT754371.1	China	2019	1
MT856318.1	China	2019	1
MT856325.1	China	2019	1
MT856330.1	China	2019	1
MW018168.1	China	2019	1
MW018169.1	China	2019	1
MW018170.1	China	2019	1
MW365269.1	China	2019	1
MZ277524.1	India	2018	1
MZ452990.1	China	2019	1
MZ452991.1	China	2019	1
MZ452993.1	China	2019	1
MZ452994.1	China	2019	1
MZ452996.1	China	2019	1
MZ453006.1	China	2019	1

MZ453008.1	China	2019	1
MZ453009.1	China	2019	1
MZ453010.1	China	2019	1
OM639980.1	India	2021	1
OM639982.1	India	2021	1
OM639992.1	India	2021	1
OM639993.1	India	2021	1
OM680963.1	India	2021	1
OP389112.1	China	2022	1
OP684200.1	China	2019	1
OP684209.1	China	2019	1
OP684210.1	China	2019	1
OP684213.1	China	2019	1
OP809582.1	India	2021	1
OP921001.1	India	2022	1
OP925879.1	China	2019	1
OQ821481.1	India	2022	1
OQ821482.1	India	2022	1
OQ928148.1	China	2019	1
OQ928149.1	China	2019	1
OR029722.1	China	2019	1
OR029728.1	China	2019	1
OR029732.1	China	2019	1
OR029734.1	China	2019	1
OR492473.1	India	2019	1
OR492475.1	India	2021	1
KX621247.1	Chaina	2015	1
OQ426766.1	VietNam	2019	1
OQ832583.1	VietNam	2019	1
OQ832620.1	VietNam	2019	1
MG895089.1	Malaysia	2015	1
MN602605.1	Srilanka	2017	1
MN955685.1	Thailand	2018	1
MW018171.1	Thailand	2019	1
MZ636774.1	Thailand	2019	1
MZ636776.1	Thailand	2019	1
MZ636777.1	Thailand	2019	1
MZ636778.1	Thailand	2019	1
MZ636788.1	Thailand	2020	1
MZ636790.1	Thailand	2020	1
MZ636793.1	Thailand	2020	1
MZ636796.1	Thailand	2020	1
MZ636804.1	Thailand	2019	1
OM978278.1	SriLanka	2020	1
OP895917.1	Maldives	2021	1
OP895918.1	Cambodia	2019	1
OQ096695.1	SriLanka	2021	1

OQ102945.1	SriLanka	2021	1
OQ102946.1	SriLanka	2021	1
KX372564.1	Australia	2015	1
LC410191.1	Thailand	2017	1
MW512387.1	Singapore	2013	1
ON229507.1	Indonesia	2020	1
ON584411.1	Indonesia	2019	1
ON584414.1	Indonesia	2019	1
ON584415.1	Indonesia	2020	1
KU517847.1	Philipines	2015	1
EPI_ISL_18571270	Bangladesh	2023	1
EPI_ISL_18571264	Bangladesh	2023	1
EPI_ISL_18571265	Bangladesh	2023	1
EPI_ISL_18571267	Bangladesh	2023	1
EPI_ISL_18571268	Bangladesh	2023	1
EPI_ISL_18571275	Bangladesh	2023	1
EPI_ISL_18571276	Bangladesh	2023	1
EPI_ISL_18571277	Bangladesh	2023	1
EPI_ISL_18571278	Bangladesh	2023	1
EPI_ISL_18477971	Bangladesh	2023	1
EPI_ISL_18477972	Bangladesh	2023	1
EPI_ISL_18477974	Bangladesh	2023	1
EPI_ISL_18477975	Bangladesh	2023	1
EPI_ISL_18477976	Bangladesh	2023	1
EPI_ISL_18477977	Bangladesh	2023	1
EPI_ISL_18477979	Bangladesh	2023	1
EPI_ISL_18477980	Bangladesh	2023	1
EPI_ISL_18477981	Bangladesh	2023	1
EPI_ISL_18477982	Bangladesh	2023	1
EPI_ISL_18571266	Bangladesh	2023	1
EPI_ISL_18571269	Bangladesh	2023	1
EPI_ISL_18571271	Bangladesh	2023	1
EPI_ISL_18571272	Bangladesh	2023	1
EPI_ISL_18571273	Bangladesh	2023	1
EPI_ISL_18571274	Bangladesh	2023	1
LC775725.1	Japan	2020	2
MN923111.1	China	2019	2
AY858035.2	Indonesia	2004	2
MW362792.1	Indonesia	2019	2
MW362793.1	Indonesia	2019	2
MN923112.1	China	2019	3
OM368351.1	China	2019	3
OP808344.1	India	2022	3
OP808345.1	India	2022	3
OP808347.1	India	2022	3
OP808348.1	India	2022	3
OP808351.1	India	2022	3

OP808352.1	India	2022	3
OP808353.1	India	2022	3
OP458512.1	VietNam	2020	3
OP458515.1	VietNam	2021	3
OP458516.1	VietNam	2020	3
OP458517.1	VietNam	2019	3
OQ028207.1	VietNam	2019	3
OQ028208.1	VietNam	2019	3
OQ028212.1	VietNam	2020	3
OQ028213.1	VietNam	2019	3
OQ028230.1	VietNam	2020	3
OQ028232.1	VietNam	2020	3
OQ832572.1	VietNam	2019	3
OQ832573.1	VietNam	2019	3
OQ832576.1	VietNam	2019	3
OQ832581.1	VietNam	2019	3
OQ832584.1	VietNam	2020	3
OQ832585.1	VietNam	2020	3
OQ832586.1	VietNam	2020	3
OQ832587.1	VietNam	2020	3
OQ832588.1	VietNam	2020	3
OQ832590.1	VietNam	2020	3
OQ832594.1	VietNam	2020	3
OQ832616.1	VietNam	2019	3
OQ832618.1	VietNam	2019	3
OQ832619.1	VietNam	2019	3
OQ832621.1	VietNam	2019	3
OQ832622.1	VietNam	2019	3
MN982899.1	Australia	2019	3
MN982900.1	Australia	2019	3
MN982901.1	Australia	2019	3
LC769364.1	Japan	2023	4
MN913513.1	China	2019	4
MN923109.1	China	2019	4
MN923119.1	China	2019	4
MN923121.1	China	2019	4
MT856317.1	China	2019	4
MT856319.1	China	2019	4
MT856320.1	China	2019	4
MT856322.1	China	2019	4
MT856323.1	China	2019	4
MW295816.1	China	2020	4
MW295818.1	China	2020	4
MW720947.1	China	2019	4
MW720949.1	China	2019	4
MW720953.1	China	2019	4
MW720955.1	China	2019	4

OM639979.1	India	2021	4
OP678009.1	China	2019	4
OP684196.1	China	2019	4
OP684201.1	China	2019	4
OP684204.1	China	2019	4
OP684205.1	China	2019	4
OP684206.1	China	2019	4
OP684208.1	China	2019	4
OP684214.1	China	2019	4
OQ653841.1	China	2022	4
OR492474.1	India	2021	4
OP090388.1	VietNam	2022	4
OP090390.1	VietNam	2022	4
OP458511.1	VietNam	2019	4
OP458514.1	VietNam	2020	4
OQ028209.1	VietNam	2020	4
OQ028210.1	VietNam	2020	4
OQ028211.1	VietNam	2020	4
OQ028214.1	VietNam	2019	4
OQ028216.1	VietNam	2022	4
OQ028217.1	VietNam	2019	4
OQ028220.1	VietNam	2019	4
OQ028225.1	VietNam	2020	4
OQ028227.1	VietNam	2019	4
MZ636771.1	Thailand	2019	4
MZ636775.1	Thailand	2019	4
MZ636779.1	Thailand	2019	4
MZ636781.1	Thailand	2019	4
MZ636783.1	Thailand	2019	4
MZ636785.1	Thailand	2020	4
MZ636792.1	Thailand	2020	4
MZ636802.1	Thailand	2019	4
MZ636803.1	Thailand	2019	4
MZ636805.1	Thailand	2019	4
OL414725.1	Cambodia	2019	4
OL414727.1	Cambodia	2019	4
OL414730.1	Cambodia	2019	4
OL414732.1	Cambodia	2020	4
OL414733.1	Cambodia	2020	4
OL414736.1	Cambodia	2020	4
OL414738.1	Cambodia	2019	4
OL414745.1	Cambodia	2019	4
OL414747.1	Cambodia	2019	4
OL414755.1	Cambodia	2020	4
OL414758.1	Cambodia	2020	4
OL414763.1	Cambodia	2019	4
OL414764.1	Cambodia	2020	4

OL435143.1	Cambodia	2020	4
ON908224.1	Cambodia	2019	4
OP811977.1	Pakistan	2022	4
OP811979.1	Pakistan	2022	4
OP811981.1	Pakistan	2022	4
OP811982.1	Pakistan	2022	4
OP811983.1	Pakistan	2022	4
OP811984.1	Pakistan	2022	4
OP898559.1	Pakistan	2022	4
OP999339.1	Cambodia	2020	4
OQ674509.1	Cambodia	2019	4
OQ678017.1	Cambodia	2019	4
OQ678059.1	Cambodia	2019	4
MW186240.1	Singapore	2019	4
MW510307.1	Singapore	2019	4
MW510308.1	Singapore	2019	4
MW510310.1	Singapore	2019	4
MW510317.1	Singapore	2019	4
MW510322.1	Singapore	2019	4
MW510326.1	Singapore	2019	4
MW510333.1	Singapore	2019	4
MW512491.1	Singapore	2019	4
MW512492.1	Singapore	2019	4
MW512493.1	Singapore	2019	4
MW512494.1	Singapore	2019	4
MW512495.1	Singapore	2019	4
OP410990.1	Singapore	2019	4
MZ636773.1	Thailand	2019	5
MZ636787.1	Thailand	2020	5
ON584413.1	Indonesia	2019	5
MN944002.1	China	2019	6
MW881533.1	China	2021	6
MN548844.1	Vanuatu	2019	6
MT799878.1	Vanuatu	2020	6
MT799879.1	Vanuatu	2020	6
ON907581.1	Philippines	2019	6
OQ821493.1	Philippines	2019	6
MN548848.1	NewCaledonia	2019	6
MN548849.1	NewCaledonia	2019	6
MN548850.1	NewCaledonia	2019	6
MT799880.1	NewCaledonia	2020	6
MT799881.1	NewCaledonia	2020	6
MK905538.1	French Polynesia	2019	6
MK905539.1	French Polynesia	2019	6
MK905542.1	French Polynesia	2019	6
MN990194.1	French Polynesia	2019	6
MN990197.1	French Polynesia	2019	6

MN990199.1	French Polynesia	2019	6
MN990201.1	French Polynesia	2019	6
MN990202.1	French Polynesia	2019	6
MN990204.1	French Polynesia	2019	6
MN548853.1	Wallis and Futuna	2019	6
MN548854.1	Wallis and Futuna	2019	6
MN548855.1	Wallis and Futuna	2019	6
LC769363.1	Japan	2023	7
MN923116.1	China	2019	7
OR125606.1	China	2023	7
OP090389.1	VietNam	2022	7
OP090392.1	VietNam	2022	7
OP090393.1	VietNam	2022	7
OP984834.1	VietNam	2022	7
ON584412.1	Indonesia	2019	7
MT799882.1	NewCaledonia	2020	7
MT705606.1	China	2019	8
MT377729.1	Indonesia	2020	8
MZ130519.1	Mozambique	2019	9
OP684198.1	China	2019	10
MW648227.1	Indonesia	2019	11
MW648228.1	Indonesia	2019	11
ON123638.1 Peru	2021		12
ON123639.1 Peru	2021		12
ON123643.1 Peru	2021		12
OR654281.1	USA	2023	12
OR654284.1	USA	2023	12
OR771116.1	USA	2023	12
OR771183.1	USA	2023	12
OR771188.1	USA	2023	12
OM791800.1	Peru	2019	12
OM791801.1	Peru	2019	12
ON123638.1	Peru	2021	12
ON123640.1	Peru	2021	12
ON123641.1	Peru	2021	12
ON123643.1	Peru	2021	12
ON123644.1	Peru	2021	12
ON634743.1	Brazil	2022	12
ON634745.1	Brazil	2022	12
ON634750.1	Brazil	2022	12
ON634755.1	Brazil	2022	12
ON634756.1	Brazil	2022	12
OP599660.1	Brazil	2022	12
OP599661.1	Brazil	2021	12
OP599671.1	Brazil	2022	12
OP599672.1	Brazil	2022	12
OP599674.1	Brazil	2022	12

OP599675.1	Brazil	2022	12
OP599676.1	Brazil	2022	12
OP599685.1	Brazil	2022	12
OP599686.1	Brazil	2022	12
OP599688.1	Brazil	2022	12
OP599689.1	Brazil	2022	12
OP599691.1	Brazil	2022	12
OP599693.1	Brazil	2022	12
OP599694.1	Brazil	2022	12
OP599698.1	Brazil	2022	12
OP599699.1	Brazil	2022	12
OP599700.1	Brazil	2022	12
OP599708.1	Brazil	2022	12
OP599709.1	Brazil	2022	12
OP599710.1	Brazil	2022	12
OP599720.1	Brazil	2022	12
OP599722.1	Brazil	2022	12
OP599723.1	Brazil	2022	12
OP599724.1	Brazil	2022	12
OP599728.1	Brazil	2022	12
OP599729.1	Brazil	2022	12
OP599731.1	Brazil	2022	12
OP599733.1	Brazil	2022	12
OP599734.1	Brazil	2022	12
OP599735.1	Brazil	2022	12
OP599736.1	Brazil	2022	12
OP599737.1	Brazil	2022	12
OP599739.1	Brazil	2022	12
OP599740.1	Brazil	2022	12
OP599741.1	Brazil	2022	12
OP599743.1	Brazil	2022	12
OP599744.1	Brazil	2022	12
OP599746.1	Brazil	2022	12
OP599747.1	Brazil	2022	12
OP599748.1	Brazil	2022	12
OP599767.1	Brazil	2022	12
OP941832.1	Brazil	2022	12
OP941833.1	Brazil	2022	12
OP941836.1	Brazil	2022	12
OP941841.1	Brazil	2022	12
OQ622206.1	Brazil	2022	12
OQ622207.1	Brazil	2022	12
OQ786047.1	Brazil	2023	12
OQ786048.1	Brazil	2023	12
OR025600.1	Brazil	2023	12
OR025670.1	Brazil	2023	12
OR025674.1	Brazil	2023	12

OR025676.1	Brazil	2023	12
OR039496.1	Brazil	2022	12
OR039498.1	Brazil	2022	12
OR039500.1	Brazil	2022	12
OR039502.1	Brazil	2022	12
OR039504.1	Brazil	2022	12
OR039505.1	Brazil	2022	12
OR039506.1	Brazil	2022	12
OR039507.1	Brazil	2022	12
OR039508.1	Brazil	2022	12
OR039510.1	Brazil	2022	12
OR039511.1	Brazil	2022	12
OR039513.1	Brazil	2022	12
OR039515.1	Brazil	2022	12
OR039516.1	Brazil	2022	12
OR039517.1	Brazil	2022	12
OR039518.1	Brazil	2023	12
OR138987.1	Brazil	2022	12
OR138988.1	Brazil	2022	12
OR138989.1	Brazil	2022	12
OR138991.1	Brazil	2022	12
OR138992.1	Brazil	2022	12
OR138993.1	Brazil	2022	12
OR138994.1	Brazil	2022	12
OR138997.1	Brazil	2022	12
MG895063.1	Malaysia	2014	12
OP599749.1	Paraguay	2022	12
OP599750.1	Paraguay	2022	12
OP599755.1	Paraguay	2022	12
OP599756.1	Paraguay	2021	12
OQ725731.1	Colombia	2022	12
OQ725732.1	Colombia	2022	12
OQ725734.1	Colombia	2022	12
OQ726072.1	Colombia	2022	12
OQ726079.1	Colombia	2022	12
OR037352.1	Colombia	2022	12
OR037353.1	Colombia	2022	12
OR037354.1	Colombia	2022	12
OR037358.1	Colombia	2022	12
OR037363.1	Colombia	2022	12
OR037366.1	Colombia	2022	12
OR037367.1	Colombia	2022	12
OR037368.1	Colombia	2022	12
OR037369.1	Colombia	2022	12
OR037371.1	Colombia	2023	12
OR037377.1	Colombia	2023	12
OR037378.1	Colombia	2023	12

OR037379.1	Colombia	2023	12
MN328061.1	Bagladesh	2019	12
OR136164.1	Indonesia	2023	12
LC436617.1	Bangladesh	2017	12
LC436620.1	Bangladesh	2017	12
LC436622.1	Bangladesh	2017	12
LC436624.1	Bangladesh	2017	12
LC436625.1	Bangladesh	2017	12
LC436627.1	Bangladesh	2017	12
LC436628.1	Bangladesh	2017	12
LC436629.1	Bangladesh	2017	12
LC436630.1	Bangladesh	2017	12
LC436632.1	Bangladesh	2017	12
LC436633.1	Bangladesh	2017	12
LC436635.1	Bangladesh	2017	12
LC436636.1	Bangladesh	2017	12
LC436639.1	Bangladesh	2017	12
LC436640.1	Bangladesh	2017	12
LC436643.1	Bangladesh	2017	12
LC436644.1	Bangladesh	2017	12
LC436645.1	Bangladesh	2017	12
LC436646.1	Bangladesh	2017	12
LC436651.1	Bangladesh	2017	12
LC436652.1	Bangladesh	2017	12
LC436653.1	Bangladesh	2017	12
LC436672.1	Bangladesh	2017	12
MN328061.1	Bangladesh	2019	12
OQ826850.1	Bangladesh	2018	12
OQ826851.1	Bangladesh	2018	12
OQ826852.1	Bangladesh	2018	12
OQ826853.1	Bangladesh	2018	12
OQ826854.1	Bangladesh	2018	12
OQ826855.1	Bangladesh	2018	12
OQ826856.1	Bangladesh	2018	12
OQ826857.1	Bangladesh	2018	12
OQ826858.1	Bangladesh	2018	12
OQ826859.1	Bangladesh	2018	12
OQ826860.1	Bangladesh	2018	12
OQ826861.1	Bangladesh	2018	12
OQ826862.1	Bangladesh	2018	12
OQ826863.1	Bangladesh	2018	12
OQ826864.1	Bangladesh	2018	12
OQ826865.1	Bangladesh	2018	12
OQ826866.1	Bangladesh	2018	12
OQ826867.1	Bangladesh	2018	12
OQ826868.1	Bangladesh	2018	12
OQ826869.1	Bangladesh	2018	12

OQ826870.1	Bangladesh	2018	12
OQ826871.1	Bangladesh	2018	12
OQ826872.1	Bangladesh	2018	12
OQ826873.1	Bangladesh	2018	12
OQ826874.1	Bangladesh	2018	12
OQ826875.1	Bangladesh	2018	12
OQ826876.1	Bangladesh	2018	12
OQ826877.1	Bangladesh	2018	12
OQ826878.1	Bangladesh	2018	12
OQ826879.1	Bangladesh	2018	12
MT705575.1	China	2019	13
OP599659.1	Brazil	2022	14
OP599679.1	Brazil	2022	14
OP599681.1	Brazil	2022	14
OP599690.1	Brazil	2022	14
OP599695.1	Brazil	2022	14
OP599696.1	Brazil	2022	14
OP599697.1	Brazil	2022	14
OP599701.1	Brazil	2022	14
OP599704.1	Brazil	2022	14
OP599706.1	Brazil	2022	14
OP599712.1	Brazil	2022	14
OP599714.1	Brazil	2022	14
OP599715.1	Brazil	2022	14
OP599716.1	Brazil	2022	14
OP599718.1	Brazil	2022	14
OP599719.1	Brazil	2022	14
OP599721.1	Brazil	2022	14
OR619404.1	Colombia	2023	15
OR619405.1	Colombia	2023	15
HM488257.1	Guam	2001	16
DQ645546.1	Taiwan	2002	16
EU179857.1	Brunei	2005	16
FJ196853.1	Chaina	2003	16
AY037116.1	Australia	1993	16
EU081177.1	Singapore	2005	16
JF327392.1	Singapore	2009	16
MT705593.1	China	2019	17
MZ453011.1	China	2019	18
MZ452995.1	China	2019	19
OP599662.1	Brazil	2022	20
OP599664.1	Brazil	2022	20
OP599666.1	Brazil	2022	20
OP599667.1	Brazil	2022	20
OP599668.1	Brazil	2022	20
OP599669.1	Brazil	2022	20
OP599670.1	Brazil	2022	20

OP599677.1	Brazil	2022	20
OP599682.1	Brazil	2022	20
OP599702.1	Brazil	2022	20
OP599703.1	Brazil	2022	20
OP599705.1	Brazil	2022	20
OP599707.1	Brazil	2022	20
OP599711.1	Brazil	2022	20
OP599713.1	Brazil	2022	20
OP599759.1	Brazil	2022	20
OP599761.1	Brazil	2022	20
OP599762.1	Brazil	2022	20
OP599763.1	Brazil	2022	20
OP599765.1	Brazil	2022	20
OP599768.1	Brazil	2022	20
OP599769.1	Brazil	2022	20
