# Supplementary material for: Dengue Virus Serotype 2 Cosmopolitan C Genotype Reemerges With a New Strain in Southwest Region of Bangladesh
Source: Transbound Emerg Dis. 2025 Mar 6;2025:8275099. doi: 10.1155/tbed/8275099 (PMC12016812; doi:10.1155/tbed/8275099)
Supplement: Supporting Information — S1 includes the dendrogram of global Cosmopolitan C sequences, while S2 and S3 contain tables presenting the global and local cMDS clusters, respectively. [file 8275099.f1.zip › S3_cluster_bangladesh (1).pdf]

Accession	Country	Year	Cluster
EPI_ISL_18477971	Bangladesh	2023	1
EPI_ISL_18477974	Bangladesh	2023	1
EPI_ISL_18477976	Bangladesh	2023	1
EPI_ISL_18477979	Bangladesh	2023	1
EPI_ISL_18477982	Bangladesh	2023	1
EPI_ISL_18571265	Bangladesh	2023	1
EPI_ISL_18571266	Bangladesh	2023	1
EPI_ISL_18571270	Bangladesh	2023	1
EPI_ISL_18571271	Bangladesh	2023	1
EPI_ISL_18571272	Bangladesh	2023	1
EPI_ISL_18571274	Bangladesh	2023	1
EPI_ISL_18571276	Bangladesh	2023	1
EPI_ISL_18571278	Bangladesh	2023	1
EPI_ISL_18477980	Bangladesh	2023	1
EPI_ISL_18477977	Bangladesh	2023	1
EPI_ISL_18571268	Bangladesh	2023	1
EPI_ISL_18571277	Bangladesh	2023	1
EPI_ISL_18477975	Bangladesh	2023	1
EPI_ISL_18571273	Bangladesh	2023	1
EPI_ISL_18571275	Bangladesh	2023	1
EPI_ISL_18477972	Bangladesh	2023	1
EPI_ISL_18571269	Bangladesh	2023	1
EPI_ISL_18477981	Bangladesh	2023	1
EPI_ISL_18571267	Bangladesh	2023	1
EPI_ISL_18571264	Bangladesh	2023	1
LC436622.1	Bangladesh	2017	2
LC436639.1	Bangladesh	2017	2
LC436646.1	Bangladesh	2017	2
OQ826852.1	Bangladesh	2018	2
OQ826859.1	Bangladesh	2018	2
OQ826860.1	Bangladesh	2018	2
OQ826877.1	Bangladesh	2018	2
LC436617.1	Bangladesh	2017	2
LC436620.1	Bangladesh	2017	2
LC436624.1	Bangladesh	2017	2
LC436625.1	Bangladesh	2017	2
LC436628.1	Bangladesh	2017	2
LC436629.1	Bangladesh	2017	2
LC436630.1	Bangladesh	2017	2
LC436633.1	Bangladesh	2017	2
LC436635.1	Bangladesh	2017	2
LC436636.1	Bangladesh	2017	2
LC436640.1	Bangladesh	2017	2
LC436643.1	Bangladesh	2017	2
LC436644.1	Bangladesh	2017	2
LC436652.1	Bangladesh	2017	2

LC436653.1	Bangladesh	2017	2
LC436672.1	Bangladesh	2017	2
MN328061.1	Bagladesh	2019	2
OQ826850.1	Bangladesh	2018	2
OQ826851.1	Bangladesh	2018	2
OQ826853.1	Bangladesh	2018	2
OQ826854.1	Bangladesh	2018	2
OQ826855.1	Bangladesh	2018	2
OQ826856.1	Bangladesh	2018	2
OQ826857.1	Bangladesh	2018	2
OQ826858.1	Bangladesh	2018	2
OQ826861.1	Bangladesh	2018	2
OQ826862.1	Bangladesh	2018	2
OQ826864.1	Bangladesh	2018	2
OQ826866.1	Bangladesh	2018	2
OQ826867.1	Bangladesh	2018	2
OQ826868.1	Bangladesh	2018	2
OQ826869.1	Bangladesh	2018	2
OQ826870.1	Bangladesh	2018	2
OQ826871.1	Bangladesh	2018	2
OQ826872.1	Bangladesh	2018	2
OQ826873.1	Bangladesh	2018	2
OQ826874.1	Bangladesh	2018	2
OQ826875.1	Bangladesh	2018	2
OQ826876.1	Bangladesh	2018	2
OQ826878.1	Bangladesh	2018	2
OQ826879.1	Bangladesh	2018	2
LC436627.1	Bangladesh	2017	2
LC436632.1	Bangladesh	2017	2
LC436645.1	Bangladesh	2017	2
LC436651.1	Bangladesh	2017	2
OQ826863.1	Bangladesh	2018	2
OQ826865.1	Bangladesh	2018	2
JN036379.1	Bangladesh	2006	3
JN036372.1	Bangladesh	2009	3
JN036373.1	Bangladesh	2009	3
JN036374.1	Bangladesh	2009	3
JN036375.1	Bangladesh	2008	3
JN036376.1	Bangladesh	2008	3
JN036378.1	Bangladesh	2007	3
JN036380.1	Bangladesh	2006	3
KT781532.1	Bangladesh	2011	3
JN036377.1	Bangladesh	2008	3
LC436650.1	Bangladesh	2017	4
LC436671.1	Bangladesh	2017	4
LC436618.1	Bangladesh	2017	4
LC436619.1	Bangladesh	2017	4

LC436670.1	Bangladesh	2017	4
