# Supplementary figures and images for: Dengue Virus Serotype 2 Cosmopolitan C Genotype Reemerges With a New Strain in Southwest Region of Bangladesh
Source: Transbound Emerg Dis. 2025 Mar 6;2025:8275099. doi: 10.1155/tbed/8275099 (PMC12016812; doi:10.1155/tbed/8275099)

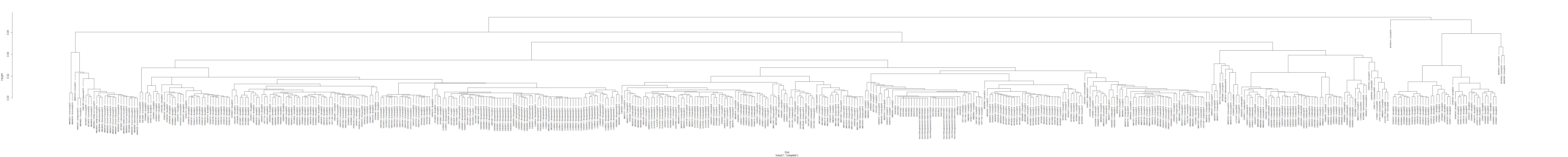

Supplement: Supporting Information — S1 includes the dendrogram of global Cosmopolitan C sequences, while S2 and S3 contain tables presenting the global and local cMDS clusters, respectively. [file 8275099.f1.zip › S1_h_clust_global_cosmopolitan_C.jpeg]
